# Contralateral Breast Background Parenchymal Enhancement: A Physiologic Marker, not a Predictor of ER Status

**DOI:** 10.2174/0115734056457319260515073941

**Published:** 2026-05-24

**Authors:** Dana Haddad, Stephanie B Shamir, Zoha Baig, Michelle Zhang, Meredith Sadinski, Min Sun Bae, Danny F Martinez, Peter Gibbs, Elizabeth A Morris, Elizabeth J Sutton

**Affiliations:** 1 American Hospital Dubai, Memorial Sloan Kettering Cancer Center, New York, USA;; 2 Montefiore Einstein Medical Center, Albert Einstein College of Medicine, New York, USA;; 3 Cornell University, Doha, Qatar;; 4 Memorial Sloan Kettering Cancer Center, New York, USA;; 5 Department of Radiology, Korea University Ansan Hospital, Korea University College of Medicine, Ansan, Republic of Korea;; 6 Department of Radiology, UC Davis School of Medicine; UC Davis Comprehensive Cancer Center, Sacramento, California, USA;; 7 Department of Diagnostic Radiology, McGill University, Montreal, Quebec, Canada

**Keywords:** Breast cancer, Background parenchymal enhancement, Estrogen receptor, BI-RADS, Tumor expression, Invasive ductal carcinoma

## Abstract

**Introduction:**

Breast MRI, specifically background parenchymal enhancement (BPE), plays a crucial role in evaluating breast cancer risk and tumor biology. BPE, influenced by vascularity, permeability, and hormonal status, reflects the physiological state of the breast tissue. This study investigates the relationship between quantitative BPE in the contralateral, non-tumor breast and estrogen receptor (ER) status in early-stage invasive ductal carcinoma (IDC), alongside factors like age, breast density, and menopausal status. It also compares quantitative BPE to qualitative BI-RADS assessments.

**Methods:**

This retrospective study included 233 women with pathologically confirmed early-stage IDC undergoing preoperative breast MRI. BPE was quantitatively assessed using MATLAB-based segmentation techniques, measuring initial, overall, late enhancement, and area under the enhancement curve (AUC). The data were compared to qualitative BI-RADS BPE categories (minimal, mild, moderate, marked). Statistical analyses were performed using Spearman’s rank correlation and Kruskal-Wallis tests, with significance set at *p*<0.05.

**Results:**

Out of 233 patients, 187 (median age 49.6, range 43.1–57.3) had ER+ cancer, while the remaining 46 (median age 52.4, range 46.9–60.6) had early-stage ER− cancer. Significant correlation between quantitative BPE values and qualitative BPE categories was observed; specifically, increasing median values of BPE enhancement curve parameters (*p*<0.001) and mean values of the top 10% of pixels (*p*<0.02) were observed across qualitative BPE categories from minimal to marked BPE. Quantitative BPE was independent of breast density (*p*>0.11), while qualitative BPE was influenced by breast density, with an increase from minimal (median=0.33, IQR=0.21–0.41) compared with marked BPE (median=0.39, IQR=0.28–0.55) (*p*=0.04). Quantitative BPE values decreased with age and menopausal status (*p*<0.02). Quantitative BPE was not associated with ER status, even when stratified by age.

**Discussion:**

The study found that quantitative BPE of the contralateral breast increased across qualitative BI-RADS BPE categories, independent of breast density, and decreased with age and post-menopausal status, but did not distinguish ER+ from ER- cancers. Published literature demonstrates age and hormone-associated effects on BPE, whereas the correlations with molecular subtype, cancer risk, and treatment response are not clear, highlighting its potential and limitations as a biomarker.

**Conclusion:**

While BPE reflects hormonal influences, its role in predicting tumor ER expression in early-stage cancer is limited. However, it provides a reproducible method for assessing breast tissue physiology, with potential utility in evaluating treatment response and hormonal effects. Furthermore, the study also delineates the need for larger, multicenter investigations to explore BPE’s potential as a complementary biomarker for breast cancer characterization and treatment response.

## 1 INTRODUCTION

Breast magnetic resonance imaging (MRI) is an established imaging modality for cancer detection, staging, and treatment monitoring [1]. For newly diagnosed patients, it is a valuable tool in the pre-operative planning by detecting lesions and is accurate in estimating disease burden [2]. Beyond the identification of lesions, MRI provides information about the normal breast microenvironment through background parenchymal enhancement (BPE) [3, 4]. As per a recent meta-analysis, the level of BPE reported on breast MRI may impact its diagnostic performance, with the mild or minimal BPE often influencing follow-ups and additional imaging to reduce missed diagnoses and unnecessary biopsies [5]. Quantitative BPE on MRI refers to the physiologic enhancement of normal fibroglandular tissue following intravenous administration of gadolinium-based contrast agents. BPE reflects vascularity, tissue permeability, and hormonal influences on breast parenchyma, thereby serving as an indirect marker of the breast’s hormonal and physiologic state. Because of these characteristics, BPE is being studied as a potential biomarker for breast cancer risk stratification, prediction of treatment response, and evaluation of tumor biology. Furthermore, the T1-weighted imaging is used for assessment of anatomical details of breast tissue, whereas the T2-weighted imaging differentiates cysts from solid masses [1, 3].

BPE, referred to as physiologic enhancement of normal fibroglandular tissue, is caused by the decrease in T1 and T2 relaxation time in breast tissue after gadolinium administration. This, in turn, increases the signal on a T1-weighted image [1, 3]. BPE is responsive to the endogenous hormonal environment, which fluctuates with various phases of the menstrual cycle, is negatively correlated with age, and is positively correlated with increasing hormonal activity [1, 4, 6]. Therefore, BPE has been studied as a tool not only for predicting breast cancer risk but also for predicting breast cancer molecular subtype and hormone receptor expression [7-9].

In addition to assessing BPE in the tumor-containing breast to predict hormone receptor expression, BPE can also be evaluated in the contralateral normal breast. This serves as a surrogate for normal tissue in the tumor-affected breast [10, 11]. Patients with early-stage human epidermal receptor-positive (HER2+) breast cancer have been found to have significantly reduced BPE in the contralateral breast following neoadjuvant chemotherapy (NAC) [11]. BPE in the contralateral breast has also been reported to be higher in patients with estrogen receptor-negative (ER−) *versus* estrogen receptor-positive (ER+) cancer, and in patients with HER2+ compared with HER2− cancer. However, statistical significance was not reached [10].

Furthermore, in clinical practice, BPE is routinely assessed qualitatively according to the Breast Imaging Reporting and Data System (BI-RADS), categorized as minimal, mild, moderate, or marked. However, qualitative grading is subject to inter- and intra-reader variability and may be confounded by radiologists’ awareness of breast density [4]. To address these limitations, fully automated quantitative segmentation methods have been developed, enabling objective and reproducible BPE measurements [12-14].

For this reason, the current study investigated the relationship between quantitative BPE in the non-tumor breast and tumor ER status in patients with early-stage breast cancer. Additionally, the study evaluated quantitative BPE in relation to breast density, age, and menopausal status, and compared it to radiologists’ qualitative BI-RADS assessments. By combining quantitative imaging analysis with established clinical parameters, the study seeks to better understand the role of BPE as a potential biomarker for breast cancer characterization.

## 2 METHODOLOGY

### 2.1 Study Design and Objectives

A retrospective study conducted from Memorial Sloan Kettering Cancer Centre, to evaluate the association between quantitative BPE of the contralateral, non-tumor breast and estrogen receptor (ER) status in women with early-stage invasive ductal carcinoma (IDC). Secondary objectives encompassed assessing the relationship between quantitative BPE and patient factors such as breast density, age, and menopausal status, as well as comparing quantitative measures with radiologists’ qualitative BPE assessments according to BI-RADS.

The study received approval (IRB number 16-512) from the institutional review board (Memorial Sloan Kettering Cancer Centre, New York, USA) and was conducted as per the Declaration of Helsinki and the International Council for Harmonization of Technical Requirements for Pharmaceuticals for Human Use guidelines. All participants provided written informed consent for the use of their data for research purposes.

### 2.2 Patient Population

Women with pathologically confirmed early-stage IDC undergoing preoperative breast MRI followed by breast-conserving surgery between 2008 and 2010 were eligible. According to the tumor-node-metastasis (TNM) classification, early-stage disease was defined as stage I or II.

Inclusion criteria: Female patients with early-stage IDC (stage I–II), availability of preoperative breast MRI, and final surgical histopathology confirming ER status.

Exclusion criteria: History of prior breast cancer and incomplete imaging or clinical data.

Eligibility criteria were fulfilled by 233 patients and included in the final analysis. Of these, 80 overlapped with a prior study that evaluated Oncotype Dx scores in relation to MRI-based imaging features [15]. ER status was determined from surgical histopathology reports. Breast density was extracted from radiologists’ assessments of mammograms using BI-RADS categories: fatty (0–25%), scattered fibroglandular (25–50%), heterogeneously dense (50–75%), and extremely dense (>75%).

### 2.3 Imaging Acquisition

MRI examinations were performed using either a 1.5 Tesla Signa scanner (n = 175; 75.1%) or a 3.0 Tesla Signa HDX scanner (n = 58, 24.9%) (GE Medical Systems, Waukesha, WI), with a dedicated 8-channel breast coil. Gadopentetate dimeglumine at a dose of 0.1 mmol/kg body weight (Magnevist; Berlex Laboratories/Bayer Health Care Pharmaceuticals, Montville, NJ) was administered to patients intravenously using an automatic injector (Medrad, Warrendale, PA) at a rate of 2 ml/s.

Sagittal T1-weighted fat-suppressed 2D multi-slice acquisitions were obtained before and sequentially three times after contrast agent administration (following a 20-second scan delay), considering parameters such as TR/TE (ms), 6.81 (range, 5.08–11.30) / 4.20 (range, 1.95–4.20); flip angle, 10° (range, 10–12), acquisition matrix, 256 × 192 (range, 256–320 × 60–256); in-plane resolution (mm), 0.78 × 0.78 (range, 0.39–0.94); slice thickness, 3 mm (range, 3–3); and temporal resolution (s), ~90. Spatial resolution and slice thickness were assumed to be constant across studies.

### 2.4 Quantitative BPE Assessment

Quantitative BPE analysis was performed in accordance with the method of Zhang *et al*. [15]. Fibroglandular tissue in the contralateral, non-tumor breast was segmented using MATLAB (Mathworks, Natick, MA). A breast radiologist (MZ) examined the first post-contrast T1-weighted image in orthogonal, sagittal, and axial maximum intensity projections, using line segmentation to separate the chest wall from breast tissue. A Gaussian smoothing filter was applied to better delineate the breast tissue from the background. An erosion filter was applied to compensate for overestimation from the smoothing filter. Patient movement during the imaging was also accounted for *via* a rigid, intensity-based registration, which was applied to both pre- and post-contrast images. Following the image augmentation, the pre-contrast T1-weighted image was used to propagate breast segmentation. The group of images was then subjected to k-means clustering for the extraction of fibroglandular tissue. Subsequently, another breast radiologist (DH) checked the completed process and manually applied adjustments where needed.

Initial enhancement was measured as the percentage increase in signal intensity from the pre-contrast (baseline) image to the first post-contrast image, compared to the baseline signal. Overall enhancement, calculated as a percentage increase in baseline signal to the final post-contrast image, was compared to the baseline signal. The percentage increase in signal from the first post-contrast image to the last post-contrast image is considered as late enhancement, using the first post-contrast image as a reference. The area under the complete contrast enhancement curve (AUC) was also calculated and normalized to the baseline signal intensity. Figure **1** shows the complete contrast enhancement curve. Pixels with negative signal changes over the entire time course were excluded. Additionally, pixels with enhancement exceeding 300% were removed, as they were considered to show non-physiologically acceptable enhancement for parenchyma.

### 2.5 Reference Standard

Qualitative BPE categories (minimal, mild, moderate, marked) assigned by radiologists according to BI-RADS were extracted from clinical imaging reports to serve as the reference standard for comparison.

### 2.6 Statistical Analysis

Pixel-level histogram-derived enhancement values were non-normally distributed and were therefore summarized using medians and interquartile ranges (IQRs). In addition, the average of the top 10% of pixels was calculated for each patient [16]. Spearman's rank correlation analysis was employed to evaluate the relationships among the four enhancement curve parameters (*i.e*., initial enhancement, overall enhancement, late enhancement, and AUC). To compare quantitative and qualitative assessments of BPE, the median values of the four enhancement curve parameters were contrasted with radiologist-assigned qualitative BPE categories (*i.e*., minimal, mild, moderate, and marked) utilizing the Kruskal–Wallis test, appropriate for non-normally distributed data.

Additionally, both quantitative BPE parameters and qualitative BPE categories were examined for their associations with breast density (computed *via* mammography images as the proportion of fibroglandular tissue relative to total breast volume) using the Kruskal-Wallis test. Lastly, differences in quantitative BPE parameters were evaluated for statistical significance through the Mann–Whitney U test. Comparisons were conducted between pre- and post-menopausal women, across six age groups (20–29, 30–39, 40–49, 50–59, 60–69, and 70+ years), and between estrogen receptor positive (ER+) and estrogen receptor negative (ER−) statuses. SPSS version 25.0 (IBM Corp., Armonk, NY) was used for all statistical analyses, with statistical significance established at *p*<0.05.

## 3 RESULT

### 3.1 Study Sample Characteristics

Out of the 233 women included in the study, 187 (median age 49.6, range 43.1–57.3) were diagnosed with ER-positive cancer, while 46 (median age 52.4, range 46.9–60.6) had ER-negative cancer. Furthermore, 134 patients (57.5%) were premenopausal, 98 (42.1%) were postmenopausal, and menopausal status was unknown for one patient. A summary of patient and tumor characteristics is provided in Table **1**.

### 3.2 Comparison of Quantitative BPE Parameters with Qualitative BPE Categories

Table **2** delineates the outcomes of the qualitative radiologist evaluation of BPE within the study cohort, alongside median values (with interquartile ranges) of quantitative BPE parameters. BPE was qualitatively characterized by radiologists as minimal in 37 (15.9%), mild in 70 (30%), moderate in 32 (13.7%), and marked in 57 (24.5%) patients. The assessment was unavailable for the remaining 37 (15.9%) patients. Initial enhancement demonstrated statistically significant differences across the qualitative BPE categories. Figure **2** depicts the box plot of initial enhancement values against each qualitative BPE category, illustrating an increase in initial enhancement values progressing from minimal to marked BPE. The box plot in Fig. (**3**) displays the AUC for each qualitative BPE category.

Statistically significant differences were observed in the median values of all four enhancement parameters, as well as in the mean of the top 10% of pixels, between the qualitative BPE categories (*p* < 0.0001 and *p* < 0.02). These values exhibited an increasing trend from minimal to marked BPE across the categories. Although individual pixels may display washout following initial enhancement, no patient exhibited washout when evaluating all fibroglandular tissue pixels collectively within the breast. Consequently, it is unsurprising that both the median values of the four-enhancement curve-based parameters (*r*>0.73, *p* < 0.001) and the means of the top 10% of pixels of these parameters (r > 0.72, *p*<0.001) demonstrated high correlation.

### 3.3 Quantitative and Qualitative BPE, and Breast Density

As depicted in Fig. (**4A**), significant differences in breast density (calculated as the proportion of fibroglandular tissue to breast volume) were noted between qualitative BPE categories (*p*=0.04), with breast density increasing across qualitative BPE categories from minimal (median=0.33, IQR 0.21–0.41) to marked BPE (median=0.39, IQR=0.28–0.55). However, as shown in the box plot in Fig. (**4B**), when breast density was compared between quantitative BPE parameters, no significant differences were observed (*p*>0.11).

### 3.4 Quantitative BPE and Age

As shown in Fig. (**5A**), we found highly significant differences in the median value for each of the four enhancement parameters, the mean of the top 10% of pixels, and AUC between age groups (*p*<0.04, 0.01, and 0.02, respectively). The data showed a clear trend of increasing parameter values up to the 30–39 age group, followed by a gradual decline in values until the 60–69 age group. However, it's worth noting that these significant differences with age disappeared when pre- and post-menopausal cases were considered separately, for both the median value of all four enhancement parameters and the average of the top 10% of pixels (*p*>0.09 for both).

### 3.5 Quantitative BPE and Menopausal Status

The box plot in Fig. (**5B**) demonstrates the various stages of enhancement observed in MRI images of the breast prior to and following menopause. Statistically significant differences were identified between pre-menopausal and post-menopausal women concerning the median values across four enhancement curves (*p* < 0.02) (Fig. **S1**). Notably, there was a marked decrease in initial, overall, and final enhancement, as well as the AUC, in post-menopausal women relative to their pre-menopausal counterparts (Fig. **S2**).

An analogous pattern was observed for the initial and final enhancement, and the AUC, when analyzing the mean of the top 10% of pixels (*p* < 0.03). Although the median of late enhancement was significantly lower in post-menopausal women (median=8.4%, IQR=6.5–11.0%) compared to pre-menopausal women (median=10.5%, IQR=7.4–13.8%), no statistically significant difference was found between the two groups concerning the mean of the top 10% of pixels (*p*=0.33).

### 3.6 Quantitative BPE and Estrogen Receptor Status

There were no significant differences in quantitative BPE between ER+ and ER- cases. To examine age-related differences, cases were grouped by age (20-29, 30-39, 40–49, 50–59, 60–69, and 70 years and above). However, the smaller age groups had too few cases for meaningful comparisons. To address this, we randomly selected cases from the larger group to match the smaller group size, repeating this process 1000 times to obtain aggregate *p-*values. This allowed us to compare the four median enhancement parameters and the four mean values of the top 10% equivalents. As shown in Fig. (**6A**-**C**) we found no correlation between these parameters for any age group (*p*=0.011 for ER+ cases, *p*=0.033 for ER- cases).

## 4 DISCUSSION

Our study demonstrated that quantitative BPE of the contralateral breast, obtained through histogram analysis performed per patient with parameters calculated pixel-by-pixel, increases across qualitative BPE categories. These categories range from minimal to marked BPE and were assessed by the radiologists in accordance with BI-RADS. While quantitative BPE of the contralateral breast was found to be independent of breast density, qualitative BPE categories were influenced by breast density. Quantitative BPE of the contralateral breast was found to decrease with age and with post-menopausal *versus* pre-menopausal status. However, quantitative BPE of the contralateral breast was not able to differentiate between ER+ and ER− breast cancer, even when patients were stratified by age.

BPE is known to be responsive to the hormonal environment [17, 18]. The current body of literature contains multiple studies that have reported that BPE decreases with age [1, 4, 6] and with post-menopausal compared to pre-menopausal status [1, 4, 6, 11, 19-21]. Our study, focusing on quantitative BPE of the contralateral breast, produced similar findings. Currently, data is sparse regarding the ability of BPE to predict molecular subtypes and hormone receptor status. Öztürk *et al*. [8] found that high BPE (combining moderate and marked categories according to the BI-RADS lexicon) in the tumor-containing breast was associated with ER+ breast cancer (*p*=0.02); and in pre-menopausal women, high BPE was also associated with molecular subtype (*p*=0.04).

Focusing on BPE of the contralateral breast, Kim *et al*. [9] found no association between ER status and BPE, similar to our results, although their study focused on BPE assessed qualitatively according to BI-RADS lexicon. They also reported that BPE of the contralateral breast was not associated with PR status or triple-negative breast cancer. Dilorenzo *et al*. [7] reported that BPE categories were distributed differently across breast cancer molecular subtypes, with mild BPE being more prevalent in luminal B cancers, while moderate and marked BPE were more prevalent in triple-negative cancers. Using a radiomics approach, Wang *et al*. reported a statistically significant association between triple-negative breast cancer and BPE heterogeneity [22].

Studies show no significant association has been found between mammographic density and BPE, similar to our study, wherein quantitative BPE was found to be independent of mammographic breast density [19, 20]. However, our results show that qualitative BPE categories seemed to be influenced by breast density, perhaps due to the radiologists’ knowledge regarding the already established relationship between increased breast cancer risk and mammographic breast density [4, 23-25]. As the degree of fibroglandular tissue (FGT) enhancement is thought to be directly related to vascular supply and permeability, it is thought that higher levels of BPE may indicate a breast tissue environment with more chances of developing breast cancer [1, 3].

The utilization of BPE as a predictive measure of breast cancer risk is crucial to the possibility of anticipating and thus preventing disease development. However, the association between baseline BPE and breast cancer risk is still debated. Baseline BPE level is associated with breast cancer risk in high-risk women [4, 26-30], but the association may not be found in women with average risk [28]. Furthermore, a study has shown a significant association between high BPE of the contralateral breast on pre-neoadjuvant chemotherapy (NAC) MRI with worse survival [31]. Moreover, a study using computer algorithm-based segmentation demonstrated that patients who achieved pathological complete response had increased pre-treatment BPE than those who did not achieve pathologic complete response [10], and another study found that a higher pretreatment BPE tended to be associated with a better response to the treatment [32].

Further adding to the controversy, there are studies that have demonstrated no significant difference in baseline BPE between a group that achieved pathologic complete response and a group that did not achieve complete response [11, 33]. While baseline BPE remains controversial, BPE does appear to have potential as an indicator of treatment response. For instance, among high-risk women carrying BRCA 1 or BRCA 2 gene receiving prophylactic risk-reducing salpingo-oophorectomy (RRSO), women without breast cancer had a decrease in BPE and FGT, while those who developed breast cancer post-RRSO tended to have higher values in all BPE and FGT measures [13]. Additionally, BPE seems to have potential as a marker of treatment response to NAC, with reductions in BPE (among women with breast cancer undergoing NAC) demonstrating better treatment outcomes [3, 10, 32, 33]. Two studies specifically reported a significant decrease in BPE (after receiving NAC) was found only in pre-menopausal, and not in post-menopausal women [21, 33].

However, when looking at the utilization of BPE for assessment of treatment response according to molecular subtype or hormone receptor status, results have been controversial. Two studies have demonstrated that NAC treatment-associated effective response may be predicted with BPE in women with HER2+ breast cancer [12, 33], while another demonstrated the absence of significant correlation between high BPE and HER2+ tumors or tumors with axillary nodal involvement [34]. Moreover, in patients having HER2-negative, ER-positive, node-negative invasive breast cancer, no association was reported between contralateral BPE and survival outcomes [35].

Future research should focus on multicenter studies incorporating standardized MRI protocols for enhancing reproducibility and external validity. Integrating quantitative BPE metrics with advanced genomic profiling and biomarkers may help in evaluating whether BPE reflects broader tumor-host interactions in addition to BPE status. Further, artificial-intelligence-based models that combine quantitative BPE with clinical and molecular variables may improve the risk stratification framework and enhance the clinical application of BPE as a complementary imaging biomarker. Clinical practice can also be enhanced using automated BPE assessment by reducing subjectivity and providing a standardized metric for risk stratification [36].

## 5 STUDY LIMITATIONS

This was a retrospective, single-center study with a modest sample size; therefore, future research should involve a multicenter study with a larger population to validate and generalize these findings across a diverse population.

## CONCLUSION

In this retrospective analysis, the quantitative background parenchymal enhancement (BPE) of the contralateral, non-tumor breast did not demonstrate predictive value for estrogen receptor (ER) status in patients with early-stage invasive ductal carcinoma. These findings remained consistent even after stratification by menopausal status and were not influenced by mammographic breast density. Importantly, while radiologist-assigned qualitative BPE categories appeared to be confounded by breast density, quantitative BPE provided a more objective and reproducible measure, reinforcing its methodological advantages over qualitative assessment. Our results contribute to the expanding body of evidence indicating that, although BPE is strongly associated with age and hormonal status, it does not reliably differentiate between ER+ and ER− cancers. This highlights the necessity for cautious interpretation of BPE within the context of tumor biology and underscores that its utility may be better suited for evaluating treatment response and hormonal influences rather than for predicting receptor status at baseline. Future large-scale, multicenter investigations that integrate quantitative BPE with advanced radiomic features and molecular profiling may elucidate its potential as a complementary imaging biomarker. Until such developments, quantitative BPE should be regarded as a promising yet limited marker of breast tissue physiology rather than a predictor of tumor ER expression.

## Figures and Tables

**Fig. (1):**
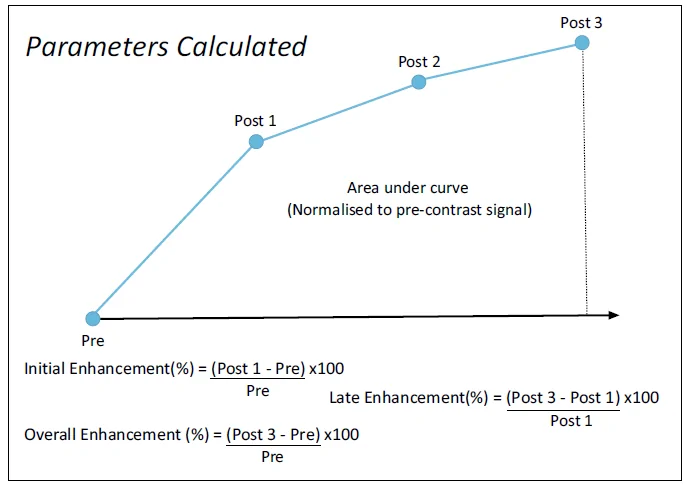
Complete contrast enhancement curve. Initial enhancement was defined as the percentage increase in signal from the pre-contrast (baseline) image to the first post-contrast image, relative to the baseline signal. Overall enhancement was defined as the percentage increase in signal from baseline to the final post-contrast image, relative to the baseline signal. Late enhancement was determined as the percentage increase in signal from the first post-contrast image to the last post-contrast image, relative to the first post-contrast signal. The area under the full contrast-enhancement curve was calculated and normalized to the baseline signal intensity.

**Fig. (2):**
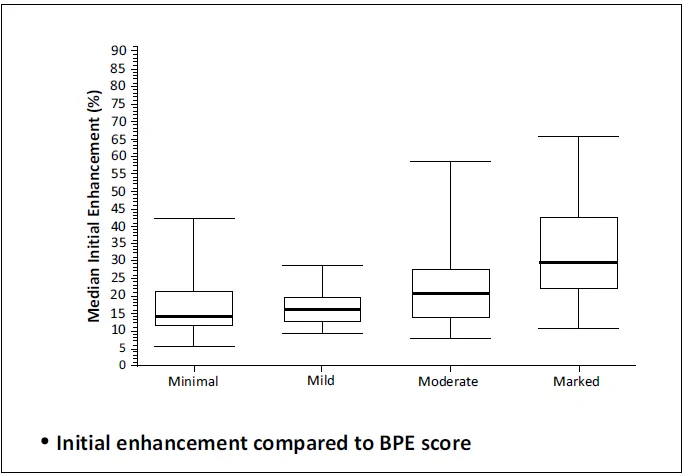
Initial enhancement median values compared with qualitative BPE category. BPE = background parenchymal enhancement.

**Fig. (3):**
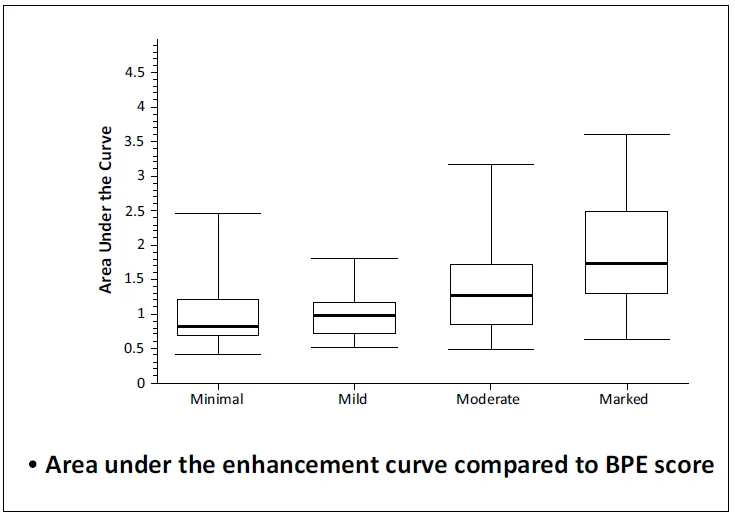
Area under the curve compared with qualitative BPE category. BPE = background parenchymal enhancement.

**Fig. (4):**
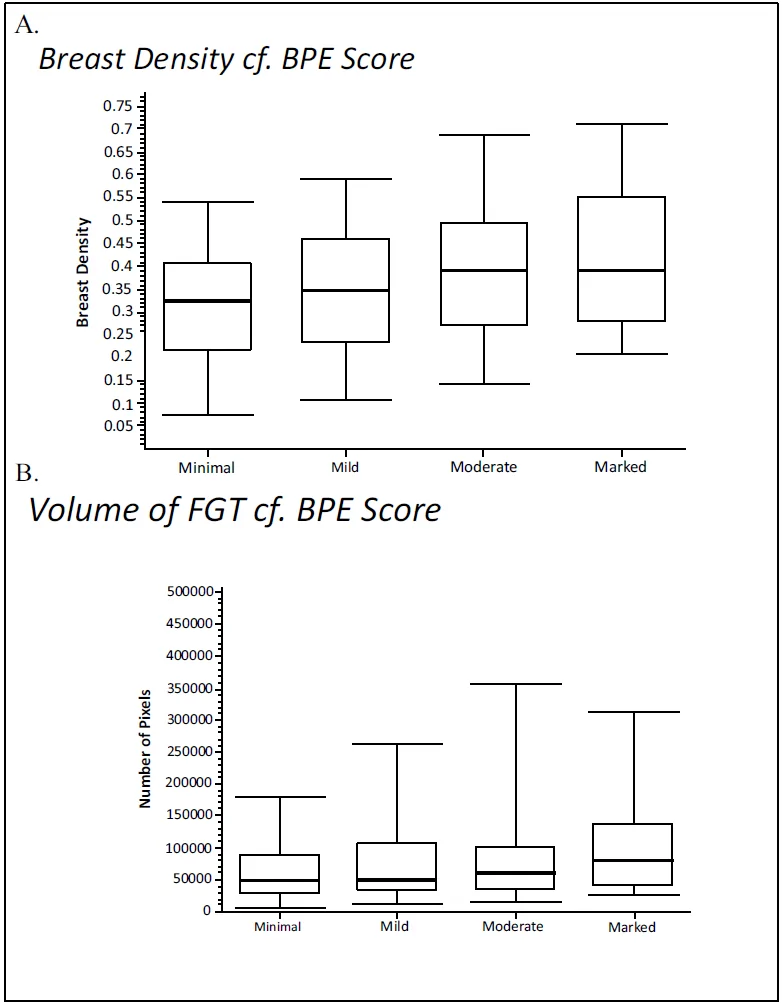
(**A**) Quantified proportion of fibroglandular tissue to breast volume between the radiologist classifications of BPE. (**B**) Proportion of fibroglandular tissue was compared to the quantitative enhancement characteristics using Spearman rank analysis. BPE = background parenchymal enhancement; FGT = fibroglandular tissue.

**Fig. (5):**
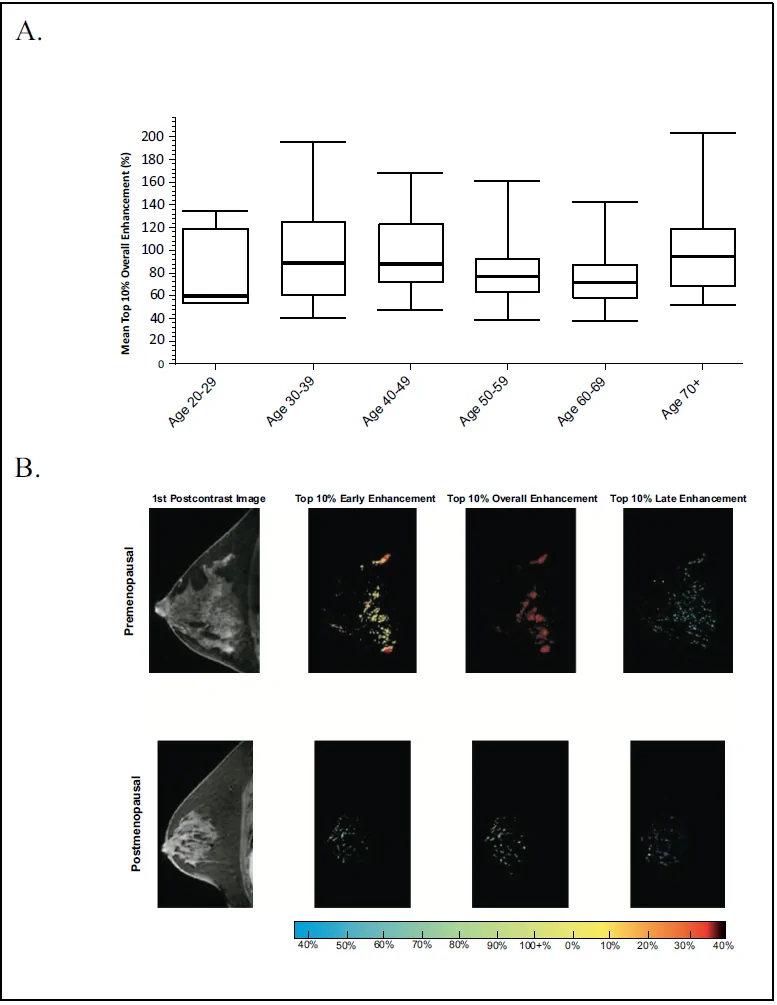
(**A**) Assessment of variation in enhancement characteristics with age for median values of all four categories of contralateral breast BPE (minimal, mild, moderate and marked) and for the mean of the top 10% of overall enhancement and area under the curve (AUC). (**B**) Visual comparison of different stages of enhancement on MR images in the pre-menopausal *vs*. post-menopausal breast.

**Fig. (6):**
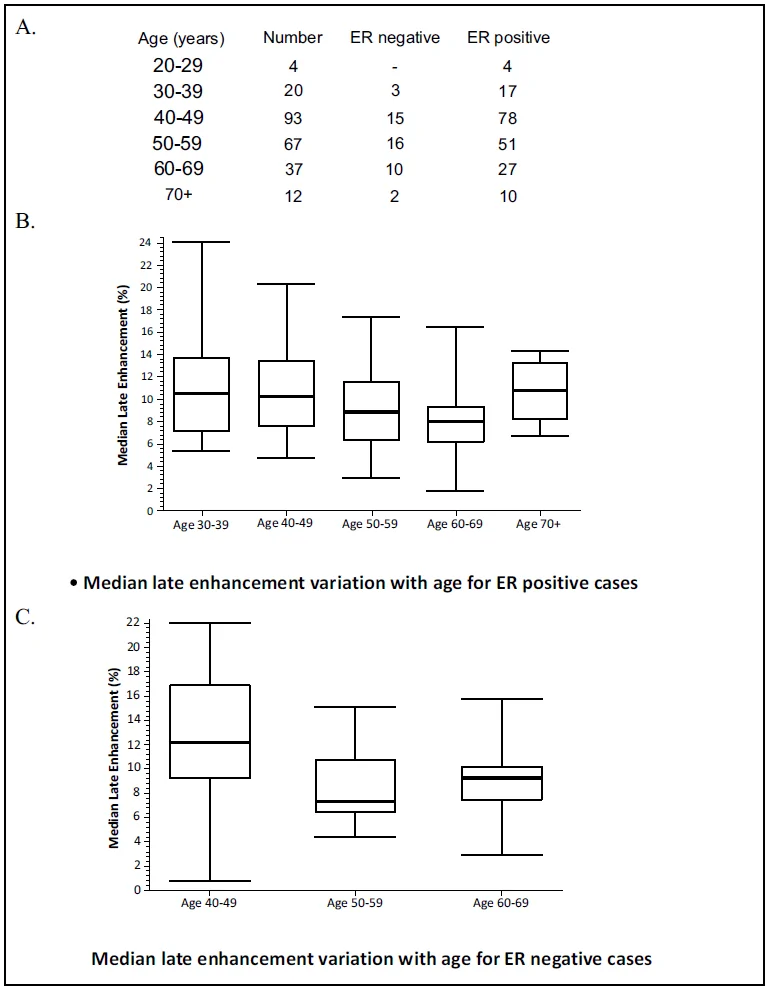
(**A**) Estrogen receptor-positive (ER+) and estrogen receptor-negative (ER−) cases. Stratified for six age bands (20–29 years, 30–39 years, 40–49 years, 50–59 years, 60–69 years, and 70+ years). Comparison of the median late enhancement values and the mean of the top 10% equivalents by (**B**) ER+ and (**C**) ER− status. No correlation was observed for ER+ or ER- patients.

**Table 1:** Demographics and molecular subtypes according to ER status.

**Categorical Variable**	**ER Negative (N = 46)** ^a^	**ER Positive (N = 187)** ^a^	***p*-value** ^b^
Age	Median (IQR)	Median (IQR)	-
-	52.4 (46.9–60.6)	49.6 (43.1–57.3)	0.07
PR Status	-	-	**<0.001**
Negative	44	19	-
Positive	2	168	-
HER2 Status	-	-	**<0.001**
Negative	31	173	-
Positive	15	14	-
Nodal Status	-	-	1.00
Negative	30	126	-
Positive	15	60	-
Unknown	1	1	-
Histology Grade	-	-	**<0.001**
1	0	18	-
2	1	62	-
3	42	100	-
Unknown	3	7	-
Nuclear Grade	-	-	**<0.001**
1	0	9	-
2	2	91	-
3	36	56	-
Unknown	8	31	-
Family History	-	-	0.62
No	21	94	-
Yes	25	93	-
Menopausal Status	-	-	0.06
Pre-menopausal	20	114	-
Post-menopausal	25	73	-
Unknown	1	0	-
HRT	-	-	1.00
No	39	161	-
Yes	6	25	-
Unknown	1	1	-
Hormone Therapy	-	-	**<0.001**
No	43	18	-
Yes	3	169	-
Radiation Therapy	-	-	0.65
No	8	28	-
Yes	38	159	-
BPE	-	-	0.55
Minimal	10	27	-
Mild	13	57	-
Moderate	4	28	-
Marked	10	47	-
Unknown	9	28	-

**Note:** ^a ^Unless otherwise indicated, values represent number of patients.^b^Comparison of characteristic between ER negatives and ER positives; listed in bold when statistically significant. **Abbreviations:** BPE = background parenchymal enhancement, ER = estrogen receptor, HER2 = human epidermal growth factor, HRT = hormone replacement therapy, IQR = interquartile range, PR = progesterone receptor.

**Table 2:** Radiological assessment of background parenchymal enhancement.

-	**Initial Enhancement (%)** ^a,b^	**Overall Enhancement (%)** ^a,b^	**Late Enhancement (%)** ^a^	**AUC^a^**
Minimal (N = 32)	46.4 (39.2–64.9)	67.7 (53.9–93.4)	27.5 (23.0–34.1)	2.46 (2.05–3.33)
Mild (N = 70)	53.7 (44.4–63.0	75.4 (61.8–87.0)	27.1 (23.7–31.9)	2.85 (2.33–3.41)
Moderate (N = 57)	64.7 (49.5–91.9)	87.2 (68.3–128.3)	29.2 (24.0–38.3)	3.34 (2.55–4.78)
Marked (N = 37)	85.7 (62.0–112.6)	120.9 (87.5–148.6)	32.9 (26.4–39.9)	4.63 (3.30–5.85)

**Note:** ^a ^Values represent medians, with interquartile ranges in parentheses.^b^Values were significantly different between groups (p<0.001)**Abbreviation:** AUC = area under the curve

## Data Availability

All the data and supportive information are provided within the article.

## LIST OF ABBREVIATIONS

AUC: Area under the curve
BI-RADS: Breast Imaging Reporting and Data System
BPE: Background parenchymal enhancement
ER: Estrogen receptor
ER−: Estrogen receptor-negative
ER+: Estrogen receptor-positive
FGT: Fibroglandular tissue
HDX: High-Definition eXtreme
HER2−: Human epidermal receptor-negative
HER2+: Human epidermal receptor-positive
IDC: Invasive ductal carcinoma
IQR: Interquartile range
MRI: Magnetic Resonance Imaging
NAC: Neoadjuvant chemotherapy
RRSO: Risk-reducing salpingo-oophorectomy
TNM: Tumor-node-metastasis

## STANDARDS OF REPORTING

STROBE guidelines were followed.

## References

[1] Hambly N.M., Liberman L., Dershaw D.D., Brennan S., Morris E.A. (2011). Background parenchymal enhancement on baseline screening breast MRI: impact on biopsy rate and short-interval follow-up.. AJR Am. J. Roentgenol..

[2] Liu Y., Gu J. (2024). Utilization of breast magnetic resonance imaging in the pre-operative setting and its ability to better predict local staging in non-metastatic breast cancer patients.. Int. Surg. J..

[3] Liao G.J., Henze Bancroft L.C., Strigel R.M., Chitalia R.D., Kontos D., Moy L., Partridge S.C., Rahbar H. (2020). Background parenchymal enhancement on breast MRI: A comprehensive review.. J. Magn. Reson. Imaging.

[4] King V., Brooks J.D., Bernstein J.L., Reiner A.S., Pike M.C., Morris E.A. (2011). Background parenchymal enhancement at breast MR imaging and breast cancer risk.. Radiology.

[5] Bechyna S., Baltzer P.A.T. (2025). Impact of background parenchymal enhancement on diagnostic performance of breast MRI: A systematic review and meta-analysis.. Radiology.

[6] Li J., Mo Y., He B., Gao Q., Luo C., Peng C., Zhao W., Ma Y., Yang Y. (2019). Association between MRI background parenchymal enhancement and lymphovascular invasion and estrogen receptor status in invasive breast cancer.. Br. J. Radiol..

[7] Dilorenzo G., Telegrafo M., La Forgia D., Stabile Ianora A.A., Moschetta M. (2019). Breast MRI background parenchymal enhancement as an imaging bridge to molecular cancer sub-type.. Eur. J. Radiol..

[8] Öztürk M., Polat A.V., Süllü Y., Tomak L., Polat A.K. (2017). Background parenchymal enhancement and fibroglandular tissue proportion on breast MRI: Correlation with hormone receptor expression and molecular subtypes of breast cancer.. J. Breast Health.

[9] Kim M.Y., Choi N., Yang J.H., Yoo Y.B., Park K.S. (2015). Background parenchymal enhancement on breast MRI and mammographic breast density: correlation with tumour characteristics.. Clin. Radiol..

[10] Chen J.H., Yu H.J., Hsu C., Mehta R.S., Carpenter P.M., Su M.Y. (2015). Background parenchymal enhancement of the contralateral normal breast: Association with tumor response in breast cancer patients receiving neoadjuvant chemotherapy.. Transl. Oncol..

[11] You C., Gu Y., Peng W., Li J., Shen X., Liu G., Peng W. (2018). Decreased background parenchymal enhancement of the contralateral breast after two cycles of neoadjuvant chemotherapy is associated with tumor response in HER2-positive breast cancer.. Acta Radiol..

[12] Nguyen A.A.T., Arasu V.A., Strand F., Li W., Onishi N., Gibbs J., Jones E.F., Joe B.N., Esserman L.J., Newitt D.C., Hylton N.M. (2020). Comparison of segmentation methods in assessing background parenchymal enhancement as a biomarker for response to neoadjuvant therapy.. Tomography.

[13] Sekine M., Nishino K., Enomoto T. (2021). BRCA genetic test and risk-reducing salpingo-oophorectomy for hereditary breast and ovarian cancer: state-of-the-art.. Cancers.

[14] Ma X., Wang J., Zheng X., Liu Z., Long W., Zhang Y., Wei J., Lu Y. (2020). Automated fibroglandular tissue segmentation in breast MRI using generative adversarial networks.. Phys. Med. Biol..

[15] Zhang M., Sadinski M., Haddad D., Bae M.S., Martinez D., Morris E.A., Gibbs P., Sutton E.J. (2021). Background parenchymal enhancement on breast MRI as a prognostic surrogate: Correlation with breast cancer Oncotype Dx score.. Front. Oncol..

[16] van der Velden B.H.M., Elias S.G., Bismeijer T., Loo C.E., Viergever M.A., Wessels L.F.A., Gilhuijs K.G.A. (2017). Complementary value of contralateral parenchymal enhancement on DCE-MRI to prognostic models and molecular assays in high-risk ER^+^/HER2^−^ breast cancer.. Clin. Cancer Res..

[17] Klifa C., Suzuki S., Aliu S., Singer L., Wilmes L., Newitt D., Joe B., Hylton N. (2011). Quantification of background enhancement in breast magnetic resonance imaging.. J. Magn. Reson. Imaging.

[18] Giess C.S., Yeh E.D., Raza S., Birdwell R.L. (2014). Background parenchymal enhancement at breast MR imaging: normal patterns, diagnostic challenges, and potential for false-positive and false-negative interpretation.. Radiographics.

[19] Kawamura A., Satake H., Ishigaki S., Ikeda M., Kimura R., Shimamoto K., Naganawa S. (2015). Prediction of background parenchymal enhancement on breast MRI using mammography, ultrasonography, and diffusion-weighted imaging.. Nagoya J. Med. Sci..

[20] Scaranelo A.M., Carrillo M.C., Fleming R., Jacks L.M., Kulkarni S.R., Crystal P. (2013). Pilot study of quantitative analysis of background enhancement on breast MR images: association with menstrual cycle and mammographic breast density.. Radiology.

[21] Chen J.H., Yu H., Lin M., Mehta R.S., Su M.Y. (2013). Background parenchymal enhancement in the contralateral normal breast of patients undergoing neoadjuvant chemotherapy measured by DCE-MRI.. Magn. Reson. Imaging.

[22] Wang J., Kato F., Oyama-Manabe N., Li R., Cui Y., Tha K.K., Yamashita H., Kudo K., Shirato H. (2015). Identifying triple-negative breast cancer using background parenchymal enhancement heterogeneity on dynamic contrast-enhanced MRI: A pilot radiomics study.. PLoS One.

[23] Boyd N.F., Guo H., Martin L.J., Sun L., Stone J., Fishell E., Jong R.A., Hislop G., Chiarelli A., Minkin S., Yaffe M.J. (2007). Mammographic density and the risk and detection of breast cancer.. N. Engl. J. Med..

[24] Vachon C.M., van Gils C.H., Sellers T.A., Ghosh K., Pruthi S., Brandt K.R., Pankratz V.S. (2007). Mammographic density, breast cancer risk and risk prediction.. Breast Cancer Res..

[25] Pike M.C., Pearce C.L. (2013). Mammographic density, MRI background parenchymal enhancement and breast cancer risk.. Ann Oncol..

[26] Dontchos B.N., Rahbar H., Partridge S.C., Korde L.A., Lam D.L., Scheel J.R., Peacock S., Lehman C.D. (2015). Are qualitative assessments of background parenchymal enhancement, amount of fibroglandular tissue on MR images, and mammographic density associated with breast cancer risk?. Radiology.

[27] Saha A., Grimm L.J., Ghate S.V., Kim C.E., Soo M.S., Yoon S.C., Mazurowski M.A. (2019). Machine learning‐based prediction of future breast cancer using algorithmically measured background parenchymal enhancement on high‐risk screening MRI.. J. Magn. Reson. Imaging.

[28] Niell B.L., Abdalah M., Stringfield O., Raghunand N., Ataya D., Gillies R., Balagurunathan Y. (2021). Quantitative measures of background parenchymal enhancement predict breast cancer risk.. AJR Am. J. Roentgenol..

[29] Grimm L.J., Saha A., Ghate S.V., Kim C., Soo M.S., Yoon S.C., Mazurowski M.A. (2019). Relationship between background parenchymal enhancement on high-risk screening MRI and future breast cancer risk.. Acad. Radiol..

[30] Thompson C.M., Mallawaarachchi I., Dwivedi D.K., Ayyappan A.P., Shokar N.K., Lakshmanaswamy R., Dwivedi A.K. (2019). The association of background parenchymal enhancement at breast MRI with breast cancer: A systematic review and meta-analysis.. Radiology.

[31] Choi J.S., Ko E.S., Ko E.Y., Han B.K., Nam S.J. (2016). Background parenchymal enhancement on preoperative magnetic resonance imaging: Association with recurrence-free survival in breast cancer patients treated with neoadjuvant chemotherapy.. Medicine.

[32] Cho H., Park M., Park H., Ko E.S., Hwang N.Y., Im Y.H., Ko K., Sim S.H. (2020). The tumor–fat interface volume of breast cancer on pretreatment mri is associated with a pathologic response to neoadjuvant chemotherapy.. Biology.

[33] Dong J.M., Wang H.X., Zhong X.F., Xu K., Bian J., Feng Y., Chen L., Zhang L., Wang X., Ma D.J., Wang B. (2018). Changes in background parenchymal enhancement in HER2-positive breast cancer before and after neoadjuvant chemotherapy.. Medicine.

[34] Ha R., Mango V., Al-Khalili R., Mema E., Friedlander L., Desperito E., Wynn R.T. (2018). Evaluation of association between degree of background parenchymal enhancement on MRI and breast cancer subtype.. Clin. Imaging.

[35] Shin G.W., Zhang Y., Kim M.J., Su M.Y., Kim E.K., Moon H.J., Yoon J.H., Park V.Y. (2018). Role of dynamic contrast‐enhanced MRI in evaluating the association between contralateral parenchymal enhancement and survival outcome in ER‐positive, HER2‐negative, node‐negative invasive breast cancer.. J. Magn. Reson. Imaging.

[36] Duong K.S., Rubner R., Siegel A., Adam R., Ha R., Maldjian T. (2024). Machine learning assessment of background parenchymal enhancement in breast cancer and clinical applications: A literature review.. Cancers.

