# Supplementary material for: Contralateral Breast Background Parenchymal Enhancement: A Physiologic Marker, not a Predictor of ER Status
Source: Curr Med Imaging. 2026 May 24;22:E15734056457319. doi: 10.2174/0115734056457319260515073941 (PMC13728981; doi:10.2174/0115734056457319260515073941)
Supplement: Supplementary file 1 [file CMIM-22-E15734056457319_SD1.pdf]

1875-6603/26 Send Orders for Reprints to reprints@benthamscience.net

BENTHAM SCIENCE
CrossMark

Current Medical Imaging

Content list available at: https://benthamscience.com/journals/cmir

Supplementary Material

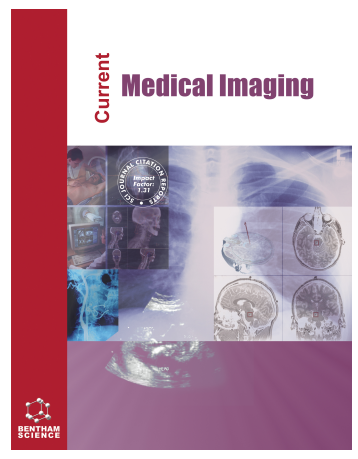

Contralateral Breast Background Parenchymal Enhancement: A Physiologic Marker, not a Predictor of ER Status

Dana Haddad[1,*], Stephanie B Shamir[2], Zoha Baig[3], Michelle Zhang[4,7], Meredith Sadinski[4], Min Sun Bae[5], Danny F Martinez[4], Peter Gibbs[4], Elizabeth A Morris[4,6] and Elizabeth J Sutton[4]

[1]*American Hospital Dubai, Memorial Sloan Kettering Cancer Center, New York, USA*
[2]*Montefiore Einstein Medical Center, Albert Einstein College of Medicine, New York, USA*
[3]*Cornell University, Doha, Qatar*
[4]*Memorial Sloan Kettering Cancer Center, New York, USA*
[5]*Department of Radiology, Korea University Ansan Hospital, Korea University College of Medicine, Ansan, Republic of Korea*
[6]*Department of Radiology, UC Davis School of Medicine; UC Davis Comprehensive Cancer Center, Sacramento, California, USA*
[7]*Department of Diagnostic Radiology, McGill University, Montreal, Quebec, Canada*

Age (years)	Number	Pre-menopausal	Post-menopausal
20 – 29	4	4	-
30 – 39	20	20	-
40 – 49	93	83	10
50 – 59	67	27	39
60 – 69	37	-	37
70+	12	-	12

Menopausal status unknown in 1 case

Fig. (S1). Patients stratified by age and pre-menopausal *versus* post-menopausal status.

DOI: 10.2174/0115734056457319260515073941, 2026, *22*, e15734056457319

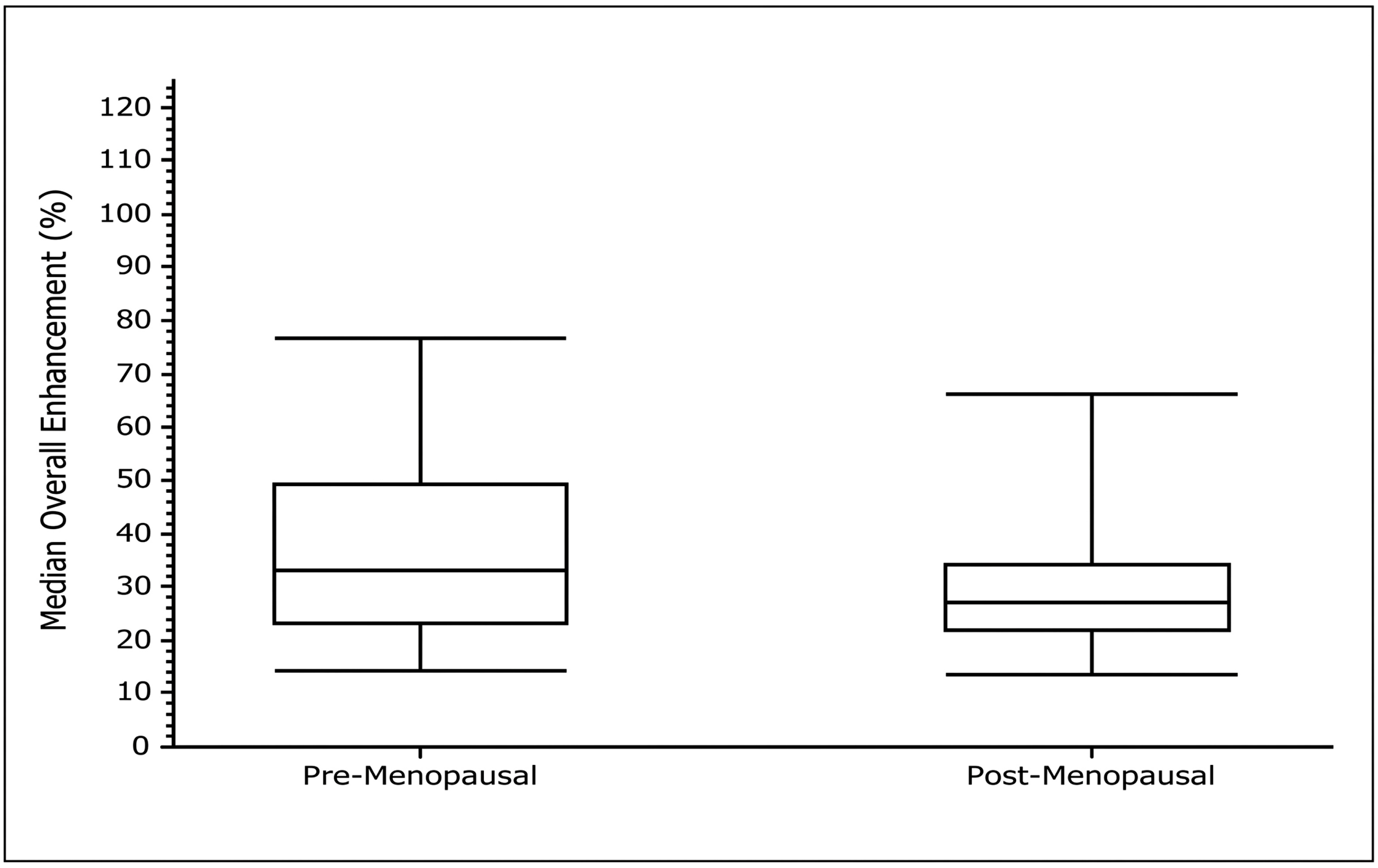

Fig. (S2). Median overall enhancement in pre-menopausal *versus* post-menopausal patients.

© 2026 The Author(s). Published by Bentham Science Publisher.

This is an open access article distributed under the terms of the Creative Commons Attribution 4.0 International Public License (CC-BY 4.0), a copy of which is available at: https://creativecommons.org/licenses/by/4.0/legalcode. This license permits unrestricted use, distribution, and reproduction in any medium, provided the original author and source are credited.

HOW TO CITE:
Haddad D, Shamir S, Baig Z, Zhang M, Sadinski M, Bae M, Martinez D, Gibbs P, Morris E, Sutton E. Contralateral Breast Background Parenchymal Enhancement: A Physiologic Marker, not a Predictor of ER Status. Curr Med Imaging, 2026; 22: e15734056457319.
http://dx.doi.org/10.2174/0115734056457319260515073941
